# 5-year follow-up of cytotoxic chemotherapy as an adjuvant to surgery in carcinoma of the bronchus.

**DOI:** 10.1038/bjc.1976.139

**Published:** 1976-08

**Authors:** H. Stott, R. J. Stephens, W. Fox, D. C. Roy

## Abstract

This report gives the 5-year findings of a double-blind study of long-term cytotoxic chemotherapy as an adjuvant to surgery in patients receiving busulphan or cyclophosphamide for carcinoma of the bronchus compared with a group receiving a placebo. Of 243 patients initially allocated busulphan, 234 cyclophosphamide and 249 placebo, 28%, 27% and 34% respectively were alive at 5 years. There were significant associations between mortality from bronchial carcinoma and histological involvement of the resected intrathoracic nodes and the histology of the tumour. Haematological toxicity, especially thrombocytopenia, was frequent and severe in the busulphan series, and low platelet counts continued long after chemotherapy was stopped.


*Br. J. Cancer* (1976) **34**, 167

### 5-YEAR FOLLOW-UP OF CYTOTOXIC CHEMOTHERAPY AS AN ADJUVANT TO SURGERY IN CARCINOMA OF THE BRONCHUS

H. STOTT, R. J. STEPHENS, W. FOX AND D. C. ROY*

*From the Medical Research Council Tuberculosis and Chest Diseases Unit, Brompton Hospital, Fulham Road, London SW3 6HP*

Received 30 March 1976 Accepted 14 April 1976

**Summary.—This report gives the 5-year findings of a double-blind study of long-term cytotoxic chemotherapy as an adjuvant to surgery in patients receiving busulphan or cyclophosphamide for carcinoma of the bronchus compared with a group receiving a placebo. Of 243 patients initially allocated busulphan, 234 cyclophosphamide and 249 placebo, 28%, 27% and 34% respectively were alive at 5 years. There were significant associations between mortality from bronchial carcinoma and histological involvement of the resected intrathoracic nodes and the histology of the tumour.**

**Haematological toxicity, especially thrombocytopenia, was frequent and severe in the busulphan series, and low platelet counts continued long after chemotherapy was stopped.**

IN 1964 a Medical Research Council Working Party planned a study to evaluate whether long-term cytotoxic chemotherapy with busulphan or cyclophosphamide, as an adjuvant to surgery in the treatment of carcinoma of the bronchus, could suppress metastases and prolong survival time, as compared with placebo. The first report (Medical Research Council Working Party, 1971) showed that up to 2 years there was no evidence that either of the cytotoxic agents conferred any benefit. There was, however, a high incidence of hazardous haematological toxicity with busulphan and of side-effects with cyclophosphamide, mostly gastrointestinal.

All the survivors have now been followed up for 5 years and the findings are reported here.

### PLAN AND CONDUCT OF THE STUDY

The plan and conduct of the study have been described in the earlier report (MRC Working Party, 1971). In brief, after total resection of the bronchial tumour and removal of all visible intrathoracic growth, the patients were allocated at random to receive tablets of busulphan (the B series), cyclophosphamide (the C series) or indistinguishable placebos (the P series) for 2 years. For the first 10 days patients received 8 tablets in 1 dose daily (1 tablet of busulphan was equivalent to 0·5 mg and 1 of cyclophosphamide to 25 mg). Thereafter for maintenance chemotherapy they received 6 tablets daily in the early stages of the study (the early intake), but due to an unexpectedly high incidence of toxicity from the cytotoxic drugs the maintenance dosages were halved to 3 tablets daily for all 3 regimens (the late intake). The study was conducted double-blind throughout the 5-year period, neither the patient nor the clinician knowing the agent allocated.

*Management of the patients.*—The patient's general condition was reported on by the physician, monthly during the first 3 years and 3-monthly thereafter, and a postero-anterior chest radiograph was taken 3-monthly. The haemoglobin estimation, total white cell and platelet counts were undertaken monthly in the first 2 years and

* Present address: Division of Pulmonary Diseases, Department of Medicine, Banaras Hindu University, Varanasi-221005, India.

thereafter only when requested by the physician.

### RESULTS

As there were no important differences between patients in the early and late intakes (except in the occurrence of drug toxicity) the amalgamated results are presented.

### *Survival in the* 3 *series up to* 5 *years*

The survival rates were similar in the 3 series (Table I and Figure). At 6 months 83% of the 243 B patients, 80% of the 234 C and 84% of the 249 P patients were alive, declining to 48%, 48% and 50% at 2 years, and 28%, 27% and 34% at 5 years. None of the differences between the series was statistically significant.

The proportions certified as dying from bronchial carcinoma during the 5 years were similar in the 3 series, 144 (59%) of the B, 142 (61%) of the C and 141 (57%) of the P patients. Patients certified as dying from other causes were 30 (12%), 29 (12%) and 23 (9%) respectively.

TABLE I.—*Survival up to 5 Years*

| | | Patients surviving at (months) | | | | | | | | | | | | | |
|---|---|---|---|---|---|---|---|---|---|---|---|---|---|---|---|
| | Total | 1 | | 6 | | 12 | | 24 | | 36 | | 48 | | 60 | |
| Treatment series | patients | No. | % | No. | % | No. | % | No. | % | No. | % | No. | % | No. | % |
| Busulphan (B) | 243 | 236 | *97* | 201 | *83* | 157 | *65* | 116 | *48* | 92 | *38* | 80 | *33* | 69 | *28* |
| Cyclophosphamide (C) | 234 | 223 | *95* | 187 | *80* | 155 | *66* | 113 | *48* | 86 | *37* | 69 | *29* | 63 | *27* |
| Placebo (P) | 249 | 238 | *96* | 208 | *84* | 163 | *65* | 124 | *50* | 105 | *42* | 97 | *39* | 85 | *34* |
| All treatments | 726 | 697 | *96* | 596 | *82* | 475 | *65* | 353 | *49* | 283 | *39* | 246 | *34* | 217 | *30* |

FIG.—Survival up to 5 years.

*Condition of the survivors at 5 years*

The general condition of the 217 survivors at 5 years was reported as good in 77%, fair in 21% and poor in 2%; 71% were at work, or, if retired, were on full activity, 27% were out and about but had restricted activity and only 5 (2%) patients were confined to hospital or bedridden. There were only minor differences between the survivors in the 3 series. Definite metastases were not reported in a single patient and were suspected in only 1 (C) patient.

*Metastases*

(a) *Frequency.*—During the 5 years, metastases were reported to be definitely present at the last examination before death in 51% of the 144 B, 42% of the 142 C and 50% of the 141 P patients who died of carcinoma of the bronchus, and were suspected in another 28%, 32% and 29%, respectively. In contrast, only 2 (1 B, 1 P) of the 83 patients whose death was certified as due to causes other than carcinoma of the bronchus had clinically definite metastases.

(b) *Time of detection.*—Within the first six months, 8% of the 243 B, 5% of the 234 C and 6% of the 249 P patients had been reported as having definite metastases; by 2 years the proportions were 25%, 21% and 25% and by 5 years 31%, 25% and 32%, respectively. Thus, the metastases appeared with similar regularity in the 3 series, none of the differences between the series being statistically significant.

*Influence of pretreatment factors on mortality from bronchial carcinoma*

Because the relationship between pretreatment factors and deaths certified as due to bronchial carcinoma were very similar in the 3 series, the results have been amalgamated in Table II. When the factors were analysed individually, there was evidence of a less favourable prognosis in females ($P = 0{\cdot}07$), in patients who had a pneumonectomy ($P = 0{\cdot}025$), in those with left-sided tumours ($P = 0{\cdot}03$)

TABLE II.—*Pretreatment Factors Related to Deaths Certified due to Bronchial Carcinoma*

| Pretreatment factor | Total patients | Patients certified dead from bronchial carcinoma No. | % |
|---|---|---|---|
| *Sex* | | | |
| Male | 670 | 387 | *58* |
| Female | 56 | 40 | *71* |
| *Operation* | | | |
| Segmental resection | 12 | 8 | *(67)* |
| Lobectomy | 339 | 183 | *54* |
| Pneumonectomy | 375 | 236 | *63* |
| *Site of tumour* (Bronchus) | | | |
| Rt Main | 18 | 8 | *(44)* |
| Rt Upper lobe | 151 | 91 | *60* |
| Rt Middle lobe | 28 | 14 | *50* |
| Rt Lower lobe | 139 | 70 | *50* |
| Lt Main | 35 | 23 | *66* |
| Lt Upper lobe | 229 | 142 | *62* |
| Lt Lower lobe | 126 | 79 | *63* |
| *Resected intrathoracic nodes* | | | |
| Involved histologically | 336 | 234 | *70* |
| Not involved histologically | 390 | 193 | *49* |
| Total patients | 726 | 427 | *59* |

and in those with histological involvement of their resected intrathoracic nodes ($P < 0{\cdot}0001$). However, a multiple stepwise regression analysis, undertaken to explore the inter-relationships of these factors, indicated that the apparent prognostic association with sex, type of operation and site of tumour was due to the influence of the histological involvement of the intrathoracic nodes. The regression analysis, by eliminating the influence of variables on each other, showed prognostic associations with histological involvement of the resected intrathoracic nodes ($P < 0{\cdot}0001$) and with histology of the tumour (epidermoid *vs.* non-epidermoid, $P = 0{\cdot}0003$) but not with age, sex, type of operation, site of tumour or treatment series.

The fatality from bronchial carcinoma of patients whose resected intrathoracic nodes were histologically involved (Table III) was higher for all 4 main tumour types than for those whose nodes were not involved, the differences for epidermoid tumours and adenocarcinoma being highly

TABLE III.—*Histological Type of Tumour according to Involvement of Intrathoracic Nodes and Deaths Certified due to Bronchial Carcinoma*

| Histological type (WHO Classification 1967) | Histology of resected intrathoracic nodes: Involved — Patients | Involved — Died from bronchial carcinoma No. | % | Not involved — Patients | Not involved — Died from bronchial carcinoma No. | % | Probability (Involved *vs.* not involved) |
|---|---|---|---|---|---|---|---|
| Epidermoid | 226 | 144 | *64* | 292 | 139 | *49* | 0·0004 |
| Large cell | 16 | 13 | *81* | 35 | 20 | *57* | NS |
| Small cell | 57 | 46 | *81* | 26 | 15 | *58* | 0·06 |
| Adenocarcinoma | 31 | 29 | *94* | 31 | 17 | *55* | 0·0016 |
| Others | 6 | 2 | (*33*) | 6 | 2 | (*33*) | NS |
| Total | 336 | 234 | *70* | 390 | 193 | *49* | 0·00001 |

significant ($P = 0{\cdot}0004$ and 0·0016, respectively). Further, the prognosis for patients with epidermoid tumours was more favourable than for those with other types of tumour; thus 64% with histological involvement of their resected nodes had died of bronchial carcinoma by 5 years compared with 81% of the patients with large cell tumours ($P = 0{\cdot}25$), 81% with small cell tumours ($P < 0{\cdot}025$) and 94% with an adenocarcinoma ($P = 0{\cdot}002$). For those whose nodes were not involved, 49% with epidermoid tumours had died, compared with 57% of those with large cell tumours, 58% with small cell tumours and 55% with adenocarcinoma, none of these differences being statistically significant.

*Drug toxicity over the 5-year period*

(a) *Deaths attributable to toxicity.*—There were 5 patients, all in the early intake in the busulphan series, in whom there was evidence that chemotherapy had materially contributed to death; four have been described in the 1971 report of the MRC Working Party. The fifth, a male of 59, died in the third year from pulmonary embolism and severe anaemia. He received busulphan daily for 7 months, a total of 610 mg; it was then interrupted because the platelet count had fallen to $69 \times 10^9/l$. After a further 60 mg of busulphan and 2 interruptions for low platelet counts it was finally stopped at 15 months when the count was $66 \times 10^9/l$. It remained low, ranging between $55 \times 10^9/l$ and $97 \times 10^9/l$ until 30 months, when a pancytopenia developed. The patient deteriorated despite repeated blood transfusions, and died in the thirty-second month without clinical evidence of metastases. A necropsy was not performed.

(b) *Haematological toxicity.*—Abnormal blood counts were reported on one or more occasions in 73% of the B compared with 37% of the C and 20% of the P patients (Table IV). The commonest manifestation on all 3 regimens was thrombocytopenia and the difference between the B patients and each of the other 2 series was highly significant ($P < 0{\cdot}0001$) for each of the 4 comparisons. Nineteen (8%) of the B patients developed a pancytopenia compared with only 1 of the C and none of the P patients.

An important feature of the thrombocytopenia in the B series was the length of time for which low platelet counts continued after the drug had been stopped. Thus in 15 (9%) the condition continued for more than 2 years, in 29 (17%) for 1 to 2 years, in 67 (39%) up to 1 year after the drug was stopped and in 61 (35%) it stopped at or before the time of termination of busulphan. However, it was not unusual for normal counts to occur sporadically among a series of abnormals. In contrast, in only 5 C and 2 P patients did abnormal platelet counts occur after stopping the tablets and in none did the condition continue for longer than 6 months.

TABLE IV.—*Incidence of Marrow Depression in the 5-year Period*

| Patients with abnormal blood counts on one or more occasions | Series: Busulphan (B) No. | % | Cyclophosphamide (C) No. | % | Placebo (P) No. | % |
|---|---|---|---|---|---|---|
| All patients | 177 | *73* | 86 | *37* | 49 | *20* |
| Thrombocytopenia (platelet count $<100 \times 10^9$/l) | 172 | *71* | 49 | *21* | 36 | *14* |
| Leucopenia (total white count $<3 \cdot 0 \times 10^9$/l) | 55 | *23* | 37 | *16* | 5 | *2* |
| Anaemia (Hb $<9$ g/dl) | 37 | *15* | 15 | *6* | 10 | *4* |
| Pancytopenia | 19 | *8* | 1 | *0* | 0 | *0* |
| Total patients | 243 | | 234 | | 249 | |

Analyses (not presented here) show that neither the total dosage of busulphan nor the size of the daily dose influenced the duration for which thrombocytopenia persisted after stopping treatment.

In view of reports (Karrer, 1972; Karrer, Pridun and Zwintz, 1973) that patients who developed leucopenia during treatment with cyclophosphamide had a better prognosis than those who did not, the occurrence of leucopenia during treatment with cyclophosphamide and with busulphan was related to survival. Twelve (32%) of 37 patients treated with cyclophosphamide who developed leucopenia were alive at 5 years compared with 51 (26%) of 197 who had no evidence of leucopenia. The corresponding proportions for patients treated with busulphan were 20 (36%) of 55 and 49 (26%) of 188 ($P = 0{\cdot}2$).

### DISCUSSION

This investigation has yielded no evidence that either of the 2 cytotoxic drugs *in the dosage schedules studied* suppressed or delayed the development of metastases in bronchial carcinoma, or improved survival in a 5-year period of observation of patients who had a total resection of their tumour and all visible intrathoracic growth and had no detectable extrathoracic metastases. There was also no evidence that either of the cytotoxic drugs influenced the survival of any subgroup of patients, identifiable by age, sex, histological involvement of resected intrathoracic nodes or type of tumour.

This study investigated 2 different cytotoxic drugs each given alone daily for a long period, a standard practice in the chemotherapy of malignant disease at the time. Since then, however, there has been some evidence that cytotoxic chemotherapy is more effective in bronchial carcinoma if given intermittently in high dosage (Bergsagel, 1971; Karrer, 1972) and if combinations of several drugs are used (Carbone *et al.*, 1970; Alberto, 1973). Most of these reports have concerned patients with limited disease or who have undergone surgical resection. Laing *et al.* (1975) however, have reported in patients with inoperable carcinoma of the bronchus that a policy of giving no immediate treatment gave better survival rates and quality of remaining life than single or multiple drug therapy.

There have been varying claims for cyclophosphamide as an adjuvant to radical surgery. Dolton (1970) reported that, given orally for 2 days before and up to 9 days after resection for carcinoma of the bronchus in 114 patients, it did not diminish the incidence of metastases or prolong survival at 2 years when compared with a previous series treated by operation only. Brunner, Marthaler and Müller (1973) found that cyclophosphamide i.v. in intermittent courses over a period of 2 years led to a significantly *increased* recurrence rate and mortality compared with an untreated control group, but Green *et al.* (1969), in a controlled study, reported improved survival of patients when the drug was administered i.v. in several courses. These studies were of all cell types but there is some evidence that cyclophosphamide is more active against small-cell tumours (Green *et al.*, 1969; Higgins, 1972; Høst, 1973). This was not the experience in the present study, nor in Tattersall and Ryall's investigation (1975) in patients treated with radiotherapy plus intermittent combination chemotherapy, including cyclophosphamide.

The patients in the present study were a selected group because all visible growth had been removed at operation, and over half had no histological involvement of the resected intrathoracic nodes. The survival of 30% at 5 years is similar to the survival rates of 25% to 30% frequently reported for bronchial carcinoma of all histological types in patients whose growth has been resected (Belcher and Anderson, 1965; Bignall, Martin and Smithers, 1967; Higgins *et al.*, 1969; Pool, 1971).

It is generally held that the best chance of survival in lung cancer is with a well-differentiated cell type and when complete resection of the growth has been possible. In the patients under study, all of whom had a radical resection, the cell type of the tumour was demonstrated to influence the prognosis only if the intrathoracic nodes were also histologically involved, patients with epidermoid tumours having a better prognosis than those with small-cell tumours ($P < 0{\cdot}05$) and those with adenocarcinoma ($P < 0{\cdot}01$). In contrast, although patients with histologically uninvolved nodes had a significantly better prognosis than those with involved nodes ($P < 0{\cdot}0001$), the differences in fatality in the various cell types in this group were small, although there was a suggestion that patients with epidermoid tumours fared slightly better.

Several other features previously reported to be of prognostic importance such as age (Belcher and Anderson, 1965; Higgins *et al.*, 1969), sex (Watson and Schottenfeld, 1968), type of operation (Bignall *et al.*, 1967; Pool, 1971) and site of tumour (Higgins and Beebe, 1967) were examined in the present study. Although associations were found with sex, type of operation and site of tumour when each was analysed individually, these were shown to be due to the influence of the other pretreatment features when examined in a multiple regression analysis, which established that the only features influencing prognosis were involvement of the intrathoracic nodes, and the histological type of growth, epidermoid tumours having a more favourable prognosis.

There was a high incidence of hazardous toxicity with busulphan, which was prescribed in a dosage which had been selected on the basis of experience in chronic myeloid leukaemia. The drug materially contributed to death in 5 (7%) of 76 patients in the early intake, and pancytopenia was exceptionally frequent, occurring in 19 (8%) of the 243 patients. A high proportion of the patients had busulphan stopped because of thrombocytopenia; many had low platelet counts for more than 1 year subsequently. In contrast, haematological abnormalities were less frequent with cyclophosphamide and seldom persisted after stopping the drug. Finally, none of the patients on busulphan (or cyclophosphamide) developed radiographic evidence of lung changes which could be ascribed to the drug (Stott *et al.*, 1976).

All the surviving patients are still being followed up to study whether there is a risk of leukaemia with either cytotoxic agent.

The surgeons, physicians and pathologists who collaborated in this study were listed in the earlier report (MRC Working Party, 1971). Their cooperation is again acknowledged and appreciated. We are particularly grateful to Dr K. F. W. Hinson for the histological typing of the tumours according to the World Health Organization Classification, 1967. Professor J. G. Scadding, the Chairman, Dr J. R. Bignall, the Secretary, and Professor

D. A. G. Galton, a member of the Working Party (disbanded in December 1972), made valuable suggestions during the preparation of this paper.

### REFERENCES

ALBERTO, P. (1973) Remission Rates, Survival, and Prognostic Factors in Combination Chemotherapy for Bronchogenic Carcinoma. *Cancer Chemother. Rep.*, **4**, 199.

BELCHER, J. R. & ANDERSON, R. (1965) Surgical Treatment of Carcinoma of the Bronchus. *Br. med. J.*, i, 948.

BERGSAGEL, D. E. (1971) An Assessment of Massive-Dose Chemotherapy of Malignant Disease. *Canad. med. Ass. J.*, **104**, 31.

BIGNALL, J. R., MARTIN, M. & SMITHERS, D. W. (1967) Survival in 6086 Cases of Bronchial Carcinoma. *Lancet*, i, 1067.

BRUNNER, K. W., MARTHALER, T. & MÜLLER, W. (1973) Effects of Long-Term Adjuvant Chemotherapy with Cyclophosphamide (NSC-26271) for Radically Resected Bronchogenic Carcinoma. *Cancer Chemother. Rep.*, **4**, 125.

CARBONE, P. P., FROST, J. K., FEINSTEIN, A. R., HIGGINS, G. A., JR. & SELAWRY, O. S. (1970) Lung Cancer: Perspectives and Prospects. *Ann. intern. Med.*, **73**, 1003.

DOLTON, E. G. (1970) Combined Surgery and Chemotherapy for Carcinoma of Bronchus. *Lancet*, i, 40.

GREEN, R. A., HUMPHREY, E., CLOSE, H. & PATNO, M. E. (1969) Alkylating Agents in Bronchogenic Carcinoma. *Am. J. Med.*, **46**, 516.

HIGGINS, G. A. & BEEBE, G. W. (1967) Bronchogenic Carcinoma—Factors in Survival. *Archs Surg.*, **94**, 539.

HIGGINS, G. A., LAWTON, R., HEILBRUNN, A. & KEEHN, R. J. (1969) Prognostic Factors in Lung Cancer—Surgical Aspects. *Ann. Thorac. Surg.*, **7**, 472.

HIGGINS, G. A. (1972) Use of Chemotherapy as an Adjuvant to Surgery for Bronchogenic Carcinoma. *Cancer N.Y.*, **30**, 1383.

HØST, H. (1973) Cyclophosphamide (NSC-26271) as Adjuvant to Radiotherapy in the Treatment of Unresectable Bronchogenic Carcinoma. *Cancer Chemother. Rep.*, **4**, 161.

KARRER, K. (1972) Importance of Dose Schedules in Adjuvant Chemotherapy. *Cancer Chemother. Rep.*, **56**, 35.

KARRER, K., PRIDUN, N. & ZWINTZ, E. (1973) Chemotherapeutic Studies in Bronchogenic Carcinoma by the Austrian Study Group. *Cancer Chemother. Rep.*, **4**, 207.

LAING, A. H., BERRY, R. J., NEWMAN, C. R. & PETO, J. (1975) Treatment of Inoperable Carcinoma of Bronchus. *Lancet*, ii, 1161.

MEDICAL RESEARCH COUNCIL WORKING PARTY (1971) Study of Cytotoxic Chemotherapy as an Adjuvant to Surgery in Carcinoma of the Bronchus. *Br. med. J.*, ii, 421.

POOL, J. L. (1971) Survival in Lung Cancer—Effectiveness of Surgery. *N.Y. State J. Med.*, **71**, 2045.

STOTT, H., STEPHENS, R., FOX, W., SIMON, G. & ROY, D. C. (1976) An Investigation of the Chest Radiographs in a Controlled Trial of Busulphan, Cyclophosphamide and a Placebo Following Resection for Carcinoma of the Lung. *Thorax*, **31**, 265.

TATTERSALL, S. & RYALL, R. D. H. (1975) Treatment of Small-Cell Carcinoma of Bronchus. *Lancet*, i, 45.

WATSON, W. L. & SCHOTTENFELD, D. (1968) Survival in Cancer of the Bronchus and Lung, 1949–1962: Comparison of Men and Women Patients. *Dis. Chest*, **53**, 65.

WORLD HEALTH ORGANIZATION (1967) *Histological Typing of Lung Tumours.* Geneva: WHO.


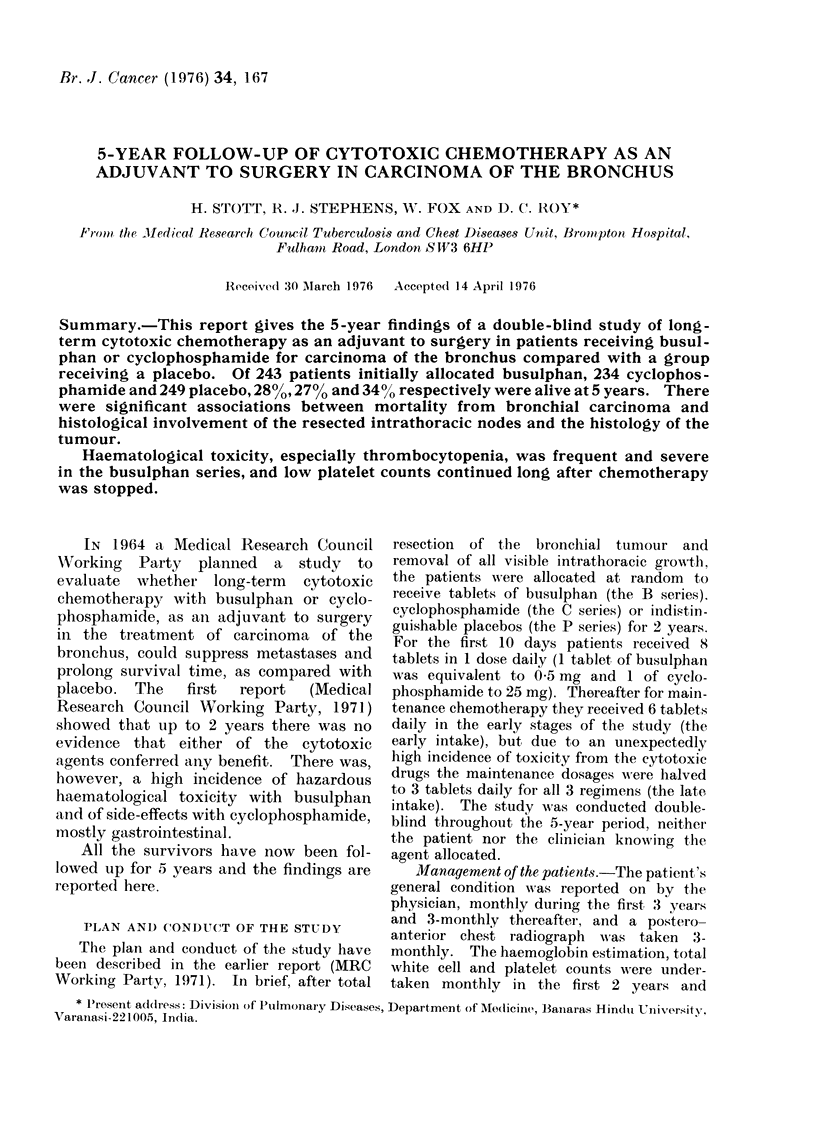

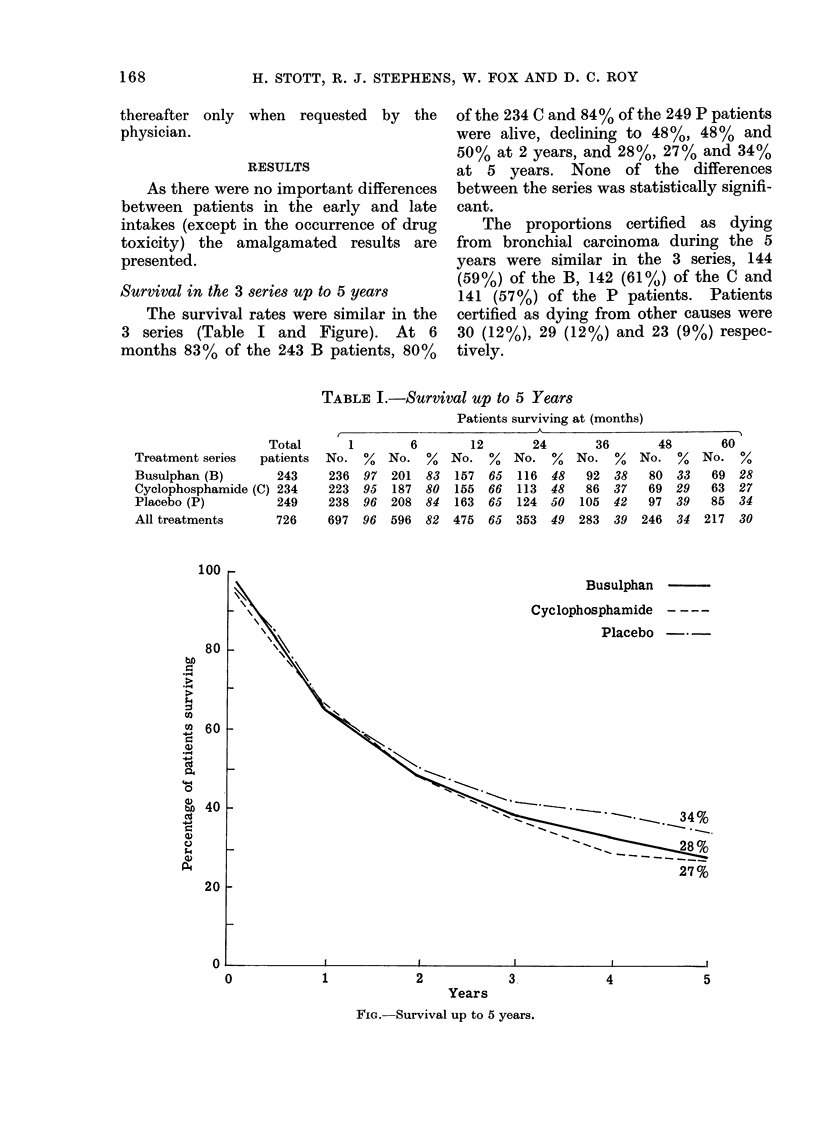

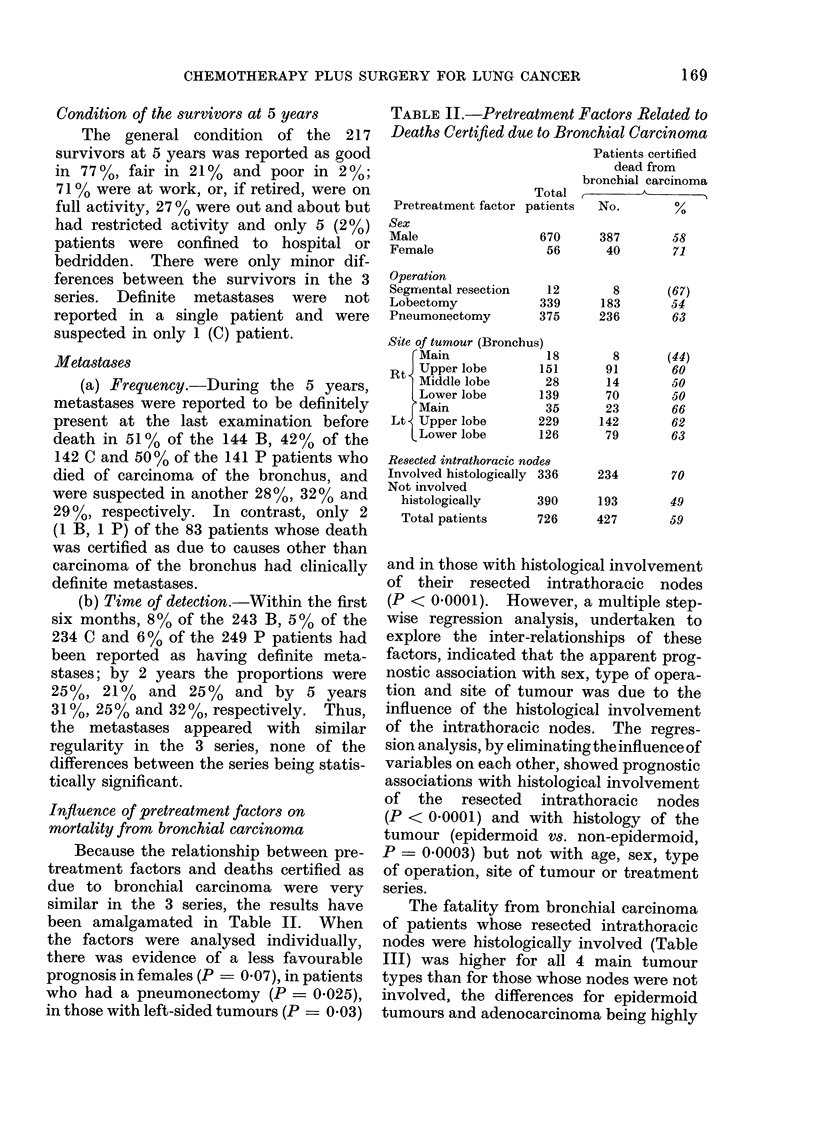

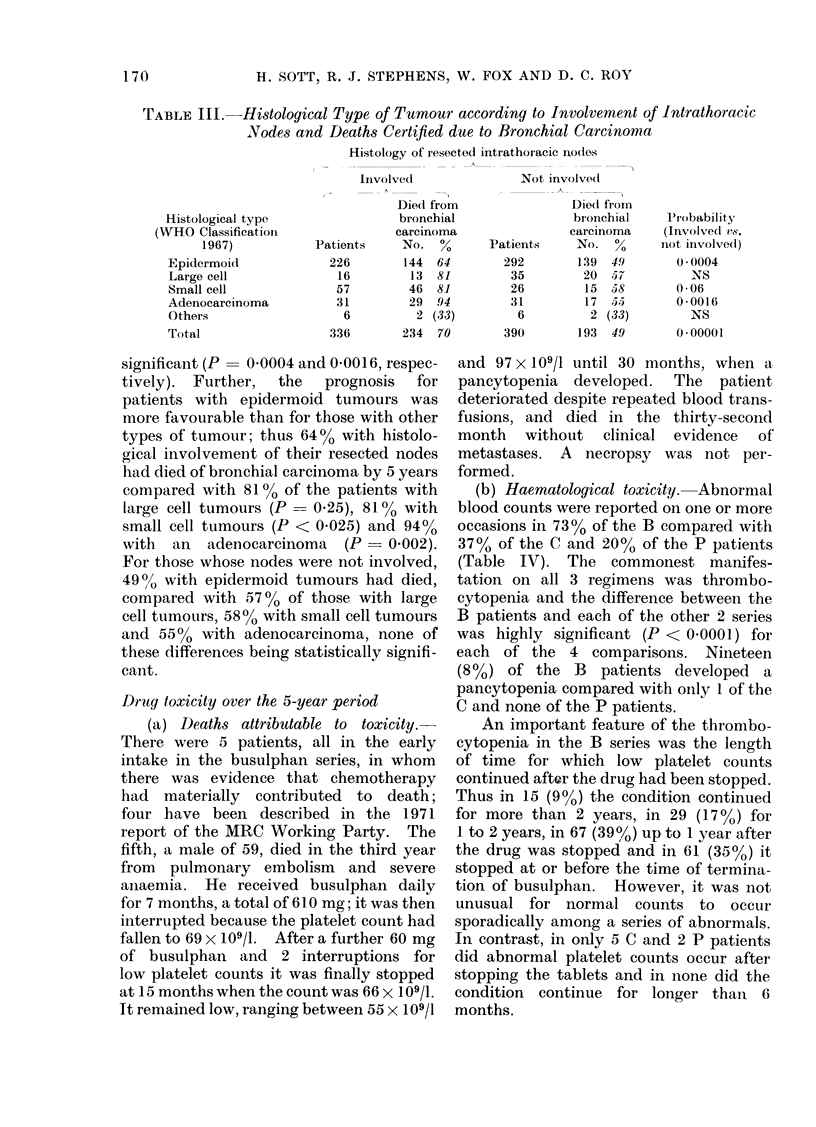

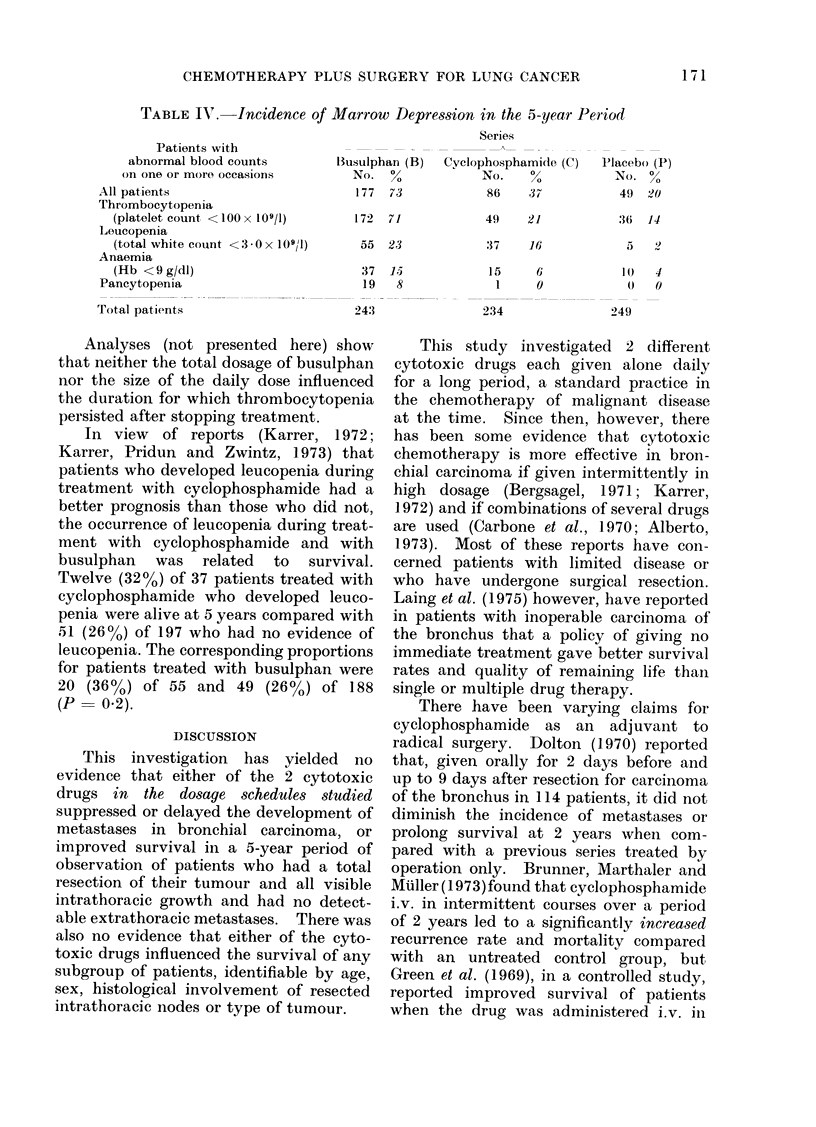

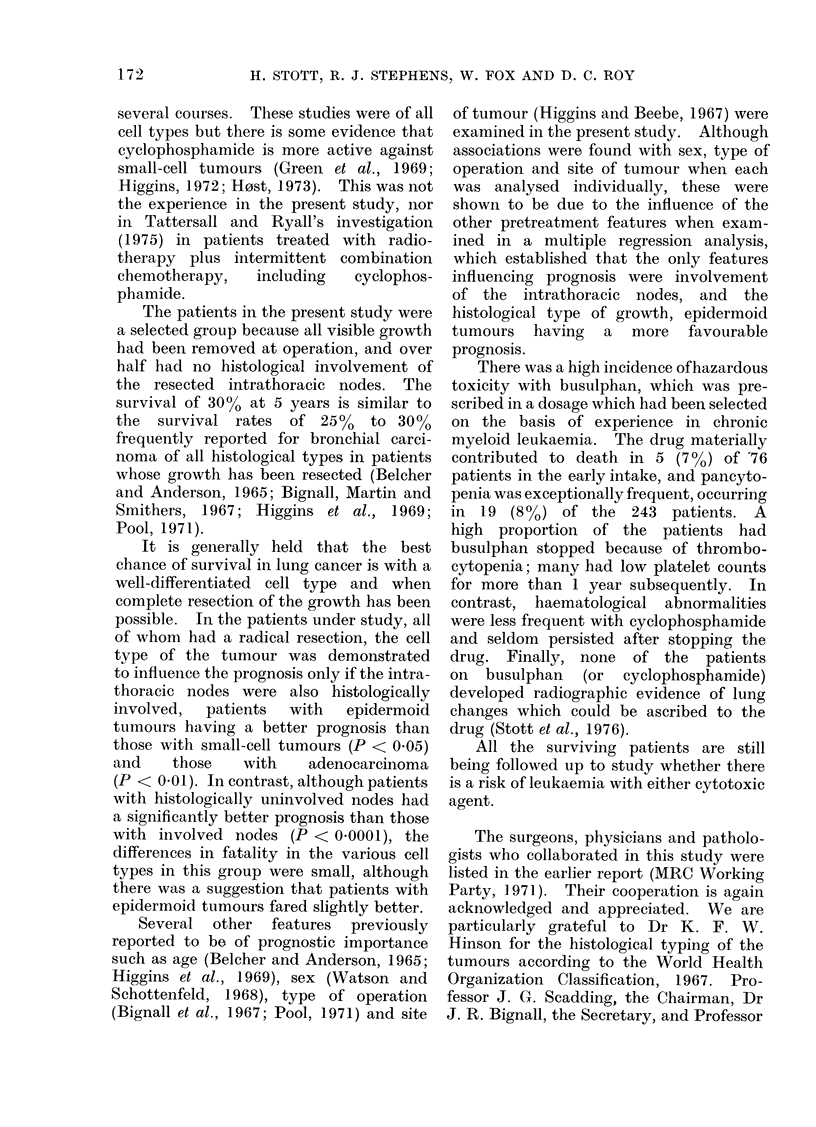

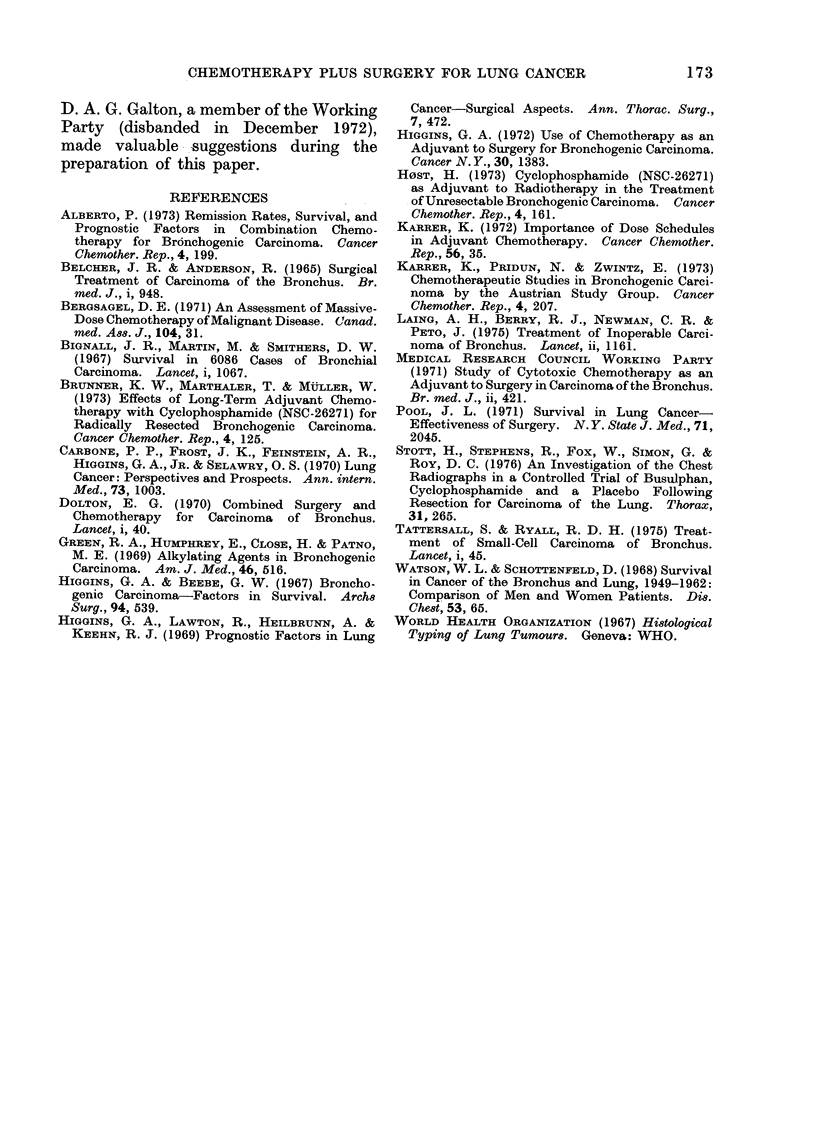


## References

[OCR_00826] Alberto P. (1973). Remission rates, survival, and prognostic factors in combination chemotherapy for bronchogenic carcinoma.. Cancer Chemother Rep 3.

[OCR_00832] BELCHER J. R., ANDERSON R. (1965). SURGICAL TREATMENT OF CARCINOMA OF THE BRONCHUS.. Br Med J.

[OCR_00837] Bergsagel D. E. (1971). An assessment of massive-dose chemotherapy of malignant disease.. Can Med Assoc J.

[OCR_00842] Bignall J. R., Martin M., Smithers D. W. (1967). Survival in 6086 cases of bronchial carcinoma.. Lancet.

[OCR_00847] Brunner K. W., Marthaler T., Müller W. (1973). Effects of long-term adjuvant chemotherapy with cyclophosphamide (NSC-26271) for radically resected bronchogenic carcinoma.. Cancer Chemother Rep 3.

[OCR_00860] Dolton E. G. (1970). Combined surgery and chemotherapy for carcinoma of bronchus.. Lancet.

[OCR_00865] Green R. A., Humphrey E., Close H., Patno M. E. (1969). Alkylating agents in bronchogenic carcinoma.. Am J Med.

[OCR_00870] Higgins G. A., Beebe G. W. (1967). Bronchogenic carcinoma. Factors in survival.. Arch Surg.

[OCR_00882] Higgins G. A. (1972). Use of chemotherapy as an adjuvant to surgery for bronchogenic carcinoma.. Cancer.

[OCR_00875] Higgins G. A., Lawton R., Heilbrunn A., Keehn R. J. (1969). Prognostic factors in lung cancer. Surgical aspects.. Ann Thorac Surg.

[OCR_00893] Karrar K. (1972). Importance of dose schedules in adjuvant chemotherapy.. Cancer Chemother Rep.

[OCR_00898] Karrer K., Pridun N., Zwintz E. (1973). Chemotherapeutic studies in bronchogenic carcinoma by the Austrian study group.. Cancer Chemother Rep 3.

[OCR_00904] Laing A. H., Berry R. J., Newman C. R., Peto J. (1975). Treatment of inoperable carcinoma of bronchus.. Lancet.

[OCR_00854] (1970). Lung cancer: perspectives and prospects.. Ann Intern Med.

[OCR_00915] Pool J. L. (1971). Survival in lung cancer; effectiveness of surgery.. N Y State J Med.

[OCR_00920] Stott H., Stephens R., Fox W., Simon G., Roy D. C. (1976). An investigation of the chest radiographs in a controlled trial of busulphan, cyclophosphamide, and a placebo after resection for carcinoma of the lung.. Thorax.

